# Maintenance of brucellosis in Yellowstone bison: linking seasonal food resources, host–pathogen interaction, and life-history trade-offs

**DOI:** 10.1002/ece3.1633

**Published:** 2015-08-20

**Authors:** John J Treanor, Chris Geremia, Michael A Ballou, Duane H Keisler, Patrick J White, John J Cox, Philip H Crowley

**Affiliations:** 1 National Park Service, Yellowstone National ParkP.O. 168, Mammoth Hot Springs, Wyoming, 82190; 2 Department of Animal and Food Sciences, Texas Tech UniversityLubbock, Texas, 79409; 3 Division of Animal Sciences, University of MissouriColumbia, Missouri, 65211; 4 Department of Forestry, University of KentuckyLexington, Kentucky, 40546; 5 Department of Biology, University of KentuckyLexington, Kentucky, 40506

**Keywords:** Age, *Brucella abortus*, condition, diet, energy, immune, interferon-*γ*, leptin, nutrition, protein

## Abstract

The seasonal availability of food resources is an important factor shaping the life-history strategies of organisms. During times of nutritional restriction, physiological trade-offs can induce periods of immune suppression, thereby increasing susceptibility to infectious disease. Our goal was to provide a conceptual framework describing how the endemic level bovine brucellosis (*Brucella abortus*) may be maintained in Yellowstone bison based on the seasonality of food resources and the life-history strategies of the host and pathogen. Our analysis was based on active *B. abortus* infection (measured via bacterial culture), nutritional indicators (measured as metabolites and hormones in plasma), and carcass measurements of 402 slaughtered bison. Data from Yellowstone bison were used to investigate (1) whether seasonal changes in diet quality affect nutritional condition and coincide with the reproductive needs of female bison; (2) whether active *B. abortus* infection and infection intensities vary with host nutrition and nutritional condition; and (3) the evidence for seasonal changes in immune responses, which may offer protection against *B. abortus*, in relation to nutritional condition. Female bison experienced a decline in nutritional condition during winter as reproductive demands of late gestation increased while forage quality and availability declined. Active *B. abortus* infection was negatively associated with bison age and nutritional condition, with the intensity of infection negatively associated with indicators of nutrition (e.g., dietary protein and energy) and body weight. Data suggest that protective cell-mediated immune responses may be reduced during the *B. abortus* transmission period, which coincides with nutritional insufficiencies and elevated reproductive demands during spring. Our results illustrate how seasonal food restriction can drive physiological trade-offs that suppress immune function and create infection and transmission opportunities for pathogens.

## Introduction

The acquisition and allocation of food resources shape the life-history strategies of organisms (Boggs 1992, 2009). During periods of food restriction, individuals may face trade-offs in the allocation of limited internal resources toward growth, maintenance, and reproduction (Stearns 1992; Zera and Harshman 2001). Consequently, investment in immune defense, which has high nutritional costs, may be reduced (Martin et al. 2008). The susceptibility to infectious diseases can increase when seasonal food insufficiencies overlap with periods of high nutritional demands (Jolly and Fernandes 2000; Buehler et al. 2010). Therefore, individuals in poor nutritional condition may be more susceptible to infection, have higher infection intensities, and serve as an important source of infection within the population (Beldomenico and Begon 2010).

The quality and availability of forage fluctuates seasonally at temperate and northern latitudes and is often limited for large mammalian herbivores during periods of high reproductive needs (Parker et al. 2009). Female ungulates frequently experience their lowest nutritional state near parturition, which can increase their susceptibility to pathogens known to establish chronic infections. Many persistent intracellular disease organisms, such as *Toxoplasma gondii*, *Coxiella burnetii*, and *Brucella abortus*, have life cycles that are timed with periods of high reproductive demands of their hosts (Innes et al. 2002; Berri et al. 2007; Carvalho Neta et al. 2010). The intracellular strategy allows these pathogens to hide within host cells until the immune system is suppressed (Rhen et al. 2003), typically when reproductive costs increase in late gestation. If reproductive demands are prioritized over immune defense, nutrient resources allocated to fetal growth and lactation may lead to a loss of immune protection near parturition (Houdijk et al. 2001; Nyman et al. 2008). In placental mammals, the breakdown of acquired immunity during energetically costly periods of reproduction can increase the probability of disease transmission, especially during the breeding season when there is a greater density of immunologically naïve young (Nelson et al. 2002; Altizer et al. 2006).

**Figure 1:**
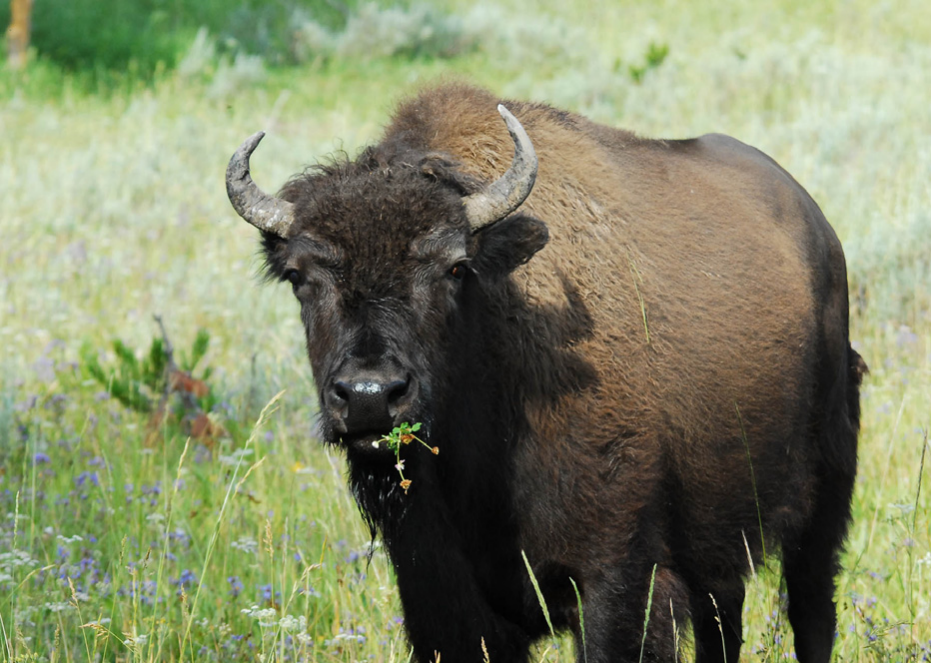
Female American bison (*Bison bison*) in Yellowstone National Park, United States (Photograph: D.S. Maehr).

**Figure 2:**
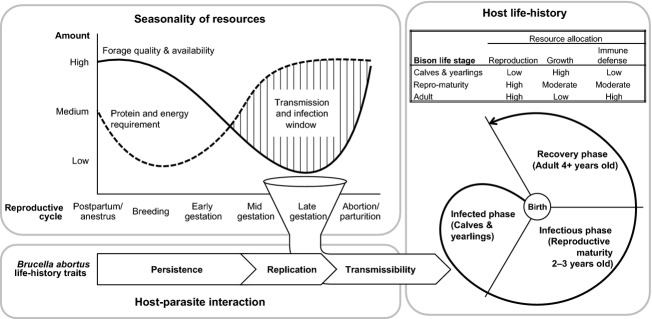
Conceptual framework describing the endemicity of brucellosis in Yellowstone bison through the integration of seasonal resource availability, host–parasite interaction, and host life-history. Forage quality declines as reproductive needs increase during late gestation, which in turn decreases nutritional condition and resources available for immune defense. The life cycle of *B. abortus* is linked with the reproductive cycle of bison to facilitate transmission when immune activity is depressed during late gestation. Seasonal food restriction influences physiological trade-offs favoring growth and reproduction over immune defense in young bison, which increase susceptibility to infection and transmission potential during pregnancy.

Yellowstone bison, the largest conservation population of plains bison (*Bison bison bison*), have been infected with the disease bovine brucellosis for nearly a century (White et al. 2015; Fig.1). We provide a conceptual framework describing how the endemic level of bovine brucellosis may be maintained in Yellowstone bison based on life-history strategies of the host and pathogen with seasonal factors influencing infection and transmission (Fig.2). *Brucella abortus* is an intracellular bacterium transmitted primarily through the ingestion of infectious tissues (e.g., fetal membranes and uterine discharges) shed from pregnant females following an abortion or at parturition (Samartino and Enright 1993). For pregnant bison, late gestation occurs during late winter and is a protein and energy demanding state as increasing demands of fetal development coincide with food insufficiency. Yellowstone bison are typically in negative energy and protein balances during winter when most forage plants are senescent and relatively low in nutritional quality; as a result, endogenous reserves (fat and body protein) are used to meet energy requirements until new vegetation growth occurs during spring (DelGiudice et al. 2001). Such seasonal reductions in protein and energy constrain immune defenses (Buehler et al. 2010) and may create opportunities for *B. abortus* transmission and infection.

The life-history traits of *B. abortus* (within-host persistence, replication rate, and transmissibility) influence bacterial fitness, and consequently, overlap with the reproductive cycle of Yellowstone bison. Investment of internal resources toward growth and winter survival may increase susceptibility to infection in immature bison, with transmission potential increasing at sexual maturity. In contrast, older bison may have lower infection and transmission rates than younger animals because they are no longer growing and have had time to recover from infection acquired earlier in life. The prevalence of brucellosis in Yellowstone bison may be linked to periods of nutritional stress and reduced immune function, both seasonally and across host life stages.

In this study, we examined how age and nutritional condition affect active brucellosis infection and the intensity of infection in Yellowstone bison. First, we assessed whether seasonal changes in diet quality affected nutritional condition and coincide with reproductive needs in female bison. Next, we tested whether active brucellosis infection and infection intensities varied with host nutrition and nutritional condition. Last, we investigated evidence for seasonal changes in immune responses, which may offer protection against *B. abortus*, in relation to nutritional condition. We hypothesized that active *B. abortus* infection and the intensity of infection would be greatest in young bison and animals in poor body condition across ages. We also expected the magnitude of protective immune responses to be greatest outside the infection and transmission window when bison are in good nutritional condition.

## Materials and Methods

### Study area and seasonal diet quality

The study was conducted in the Yellowstone National Park (Yellowstone) in the western United States during the winter of 2008, as described in Treanor et al. (2011). Yellowstone encompasses about 898,000 ha within the states of Wyoming, Montana, and Idaho, of which >90,000 ha are considered suitable habitat for bison. The bison population is divided into two separate breeding herds. The central herd occupies the central plateau and the northern herd occupies much of Yellowstone's northern boundary area. The park's geologic processes have created a range in elevations that exert strong control over the distribution of plant species (Marston and Anderson 1991). Plant communities range from grasslands and shrubs at low elevations, coniferous forest at mid-elevations, and alpine tundra near peak elevations. Winter ranges for both bison herds consist of large valley bottoms and open meadows comprised of bunch grasses and sagebrush (*Artemisia tridentata*) bordered by coniferous forest. Yellowstone winters are often severe, with snow water equivalents (i.e., mean water content of a column of snow) averaging 35 cm annually and temperatures reaching −42°C. Bison are generalist grazers, and their diet is mainly composed of graminoids (grasses and sedges). Winter conditions (e.g., deep snow pack) reduce foraging opportunities within the park, which induces migrations out of the park into some areas where bison are not tolerated because of the risk of brucellosis transmission to privately owned domestic cattle (USDI & USDA 2000; Geremia et al. 2011).

We collected bison fecal samples each month during April 2006 through September 2007 to assess seasonal changes in diet quality. Sampling was determined by the distribution of bison on sampling days. We located bison groups (>20 individuals) consisting of adult females and associated young across seasonal ranges. For each group sampled, approximately 5 fecal samples of equal volume were collected from individual bison and pooled to make a single composite sample (*n *=* *143 composite samples) for diet quality analysis. Freshly deposited fecal samples were collected into sterile Whirl-Pak® bags (Nasco, Fort Atkinson, WI) and stored frozen (−20°C). Samples were analyzed for crude protein (CP) and digestible organic matter (DOM) using near infrared reflectance spectroscopy (NIRS). The NIRS technique is an effective method for assessing diet quality in livestock and wildlife (Showers et al. 2006; Li et al. 2007; Rothman et al. 2009).

### Bison sampling

During the winter and spring (February–April) of 2008, 1434 Yellowstone bison were captured and shipped in trailers to slaughter facilities in Montana and Idaho. We collected tissue samples immediately postmortem from 402 of these bison for culture of *B. abortus*. Ages were determined by incisor eruption patterns in young bison (<5 years old; Fuller 1959) and cementum annuli analysis of the first incisor for older bison (≥5 years old) with all permanent teeth. Pregnant females were sampled during the third trimester of pregnancy, with pregnancy status determined by the presence of a fetus in the uterus. Depending on bison sex and age, we collected two sections of mammary gland and pairs of the following lymph nodes, which have been identified as the priority tissues for isolating *B. abortus* from Yellowstone bison (Rhyan et al. 2001): (1) retropharyngeal; (2) supramammary; (3) superficial inguinal; and (4) internal iliac. Tissues were collected into sterile Whirl-Pak® bags and stored frozen (−20°C) before being sent to the National Veterinary Services Laboratories (Ames, IA). Each individual tissue was analyzed separately. *Brucella abortus* isolation from tissue samples was conducted according to established standard operating procedures (USDA-NVSL a[Bibr b71]), with the suspension of each tissue plated onto five different plates. Plates were observed at 5 and 10 days for the presence of morphologically suspect *Brucella* colonies. For each plate, suspect colonies were identified as *B. abortus* using traditional biochemical analysis (USDA-NVSL b[Bibr b72]).

### Indicators of nutrition, nutritional condition, and immune function

For large herbivores, nutrition refers to the assimilation rate of ingested energy and nutrients, while nutritional condition describes the varying state of an animal's fat reserves and muscle mass that ultimately result in future fitness (Stephenson et al. 2002). During periods of food insufficiency, body reserves are mobilized resulting in metabolic changes that can be measured as adjustments in blood concentrations of hormones and metabolites (Ingvartsen and Friggens 2005). For each bison, whole blood was collected into 10-mL vacutainer tubes containing 15% EDTA (K_3_). Blood was centrifuged within 3 h after collection, and separated plasma was stored frozen (−80°C). Concentrations of plasma nonesterified fatty acids (NEFA, *n *=* *352) and urea nitrogen (BUN, *n *=* *374) were measured enzymatically in duplicate (Ballou et al. 2008). Plasma concentrations of insulin-like growth factor 1 (IGF-1, *n *=* *328) and leptin (*n *=* *326) were analyzed in triplicate by radioimmunoassay procedures (Tollefson et al. 2010) and validated for bison plasma and serum.

Nutritional condition was assessed using live weight, back fat measurements, and body condition scoring for each age group. The live weights (kg) of bison (*n *=* *262) were recorded at the Stephens Creek capture facility near Yellowstone's northern boundary using an electronic platform scale before the animals were shipped to slaughter facilities. All slaughtered bison were skinned and eviscerated, with carcasses split along the midline (midsagittal plane). Subcutaneous back fat (mm) was measured at the thickest point on carcasses just posterior to the last sacral vertebra. Carcass weights (kg, *n = *214) were measured on processed bison without hide, head, blood, internal organs, and feet. Because multiple bison were being processed simultaneously, a modified Kistner score was used to efficiently evaluate the physical condition of carcasses (Kistner et al. 1980). Body condition scores were determined based on the distribution of subcutaneous fat on the carcass and visceral fat associated with internal organs. Subjective fat indices were rated on a scale from 0 to 5 with 0.5 increments. Scores of three subcutaneous indicator sites (rump, hump, and brisket) and three visceral locations (omentum, cardiac, and perirenal) were used to produce a mean score for each bison. Each of the six sites was scored as 0–1 (little to no fat), 2–3 (moderate amount of fat), or 4–5 (abundant fat).

Cell-mediated immune responses are essential for host defense against *B. abortus*, with a bias toward T_helper_1 responses, importantly characterized by the production of interferon-*γ* (IFN-*γ*) (Waters et al. 2002; Clapp et al. 2011). Seasonal changes in IFN-*γ* were assessed from bison captured during 2007–2009 as described in Clapp et al. (2011). Because different capture methods could affect results, the seasonal production of IFN-*γ* was measured only from bison that were chemically immobilized in the field. Blood samples were collected from female bison (*n *=* *40, ages 1–17) immobilized with carfentanil and xylazine remotely administered using tranquilizing darts as part of bison management and monitoring. Blood was transferred from the jugular vein into 10-mL vacutainer tubes containing sodium heparin, refrigerated, and processed within 24 h after collection. Peripheral blood mononuclear cells (PBMC) were isolated using density gradient centrifugation, with PBMC at 5 × 10^6^ cells/mL in medium aliquoted into 24-well tissue plates for cell culture. Duplicate cultures were stimulated with concanavalin A (ConA, 1 *μ*g/mL) or unstimulated (medium only). Con A triggers the proliferation of T lymphocytes (Palacios 1982), which increases IFN-*γ* production to measurable levels. We incubated cultures at 37°C with 5% CO_2_ for 5 days and stored at −80°C until analysis. Interferon-*γ* in culture supernatants was measured using a bovine capture ELISA, with concentrations determined by comparing absorbances of test samples with absorbances of standards within a linear curve fit.

### Statistical analysis

Generalized linear models were used to evaluate the effects of age-specific nutrition and nutritional condition on active infection and infection intensity. The data for bison age were standardized by subtracting and dividing each value by the mean. Covariates for all analyses were also scaled with respect to bison age. Because condition indicators may be correlated with age, these transformations standardized covariates for each age group as the percentage above or below the mean, which facilitated interpretation of covariate effects.

To evaluate active infection in female bison (*n *=* *152), culture status was defined as a binary response variable based on the presence of at least one colony-forming unit of *Brucella abortus* on a single culture plate for all tissues assayed. The inverse-logit transformation was used to relate the probability of observing a bison with positive *B. abortus* culture status to covariates for age and nutritional condition. Animals ≥8 years of age were grouped into a single old-adult age class. A suite of 11 logistic models was developed to represent alternative hypotheses of bison age and the effect of nutritional condition on active infection. In a previous study, the relationship between active *B. abortus* infection and age demonstrated a hump-shaped pattern, with active infection peaking at sexual maturation in female bison (Treanor et al. 2011). Therefore, we included a quadratic term for age effects in all models in the current study. To evaluate the effect of nutritional condition on active *B. abortus* infection, the model suite included all combinations of age, the quadratic effect of age, live weight, and back fat thickness. We expected bison live weight and back fat thickness to be negatively associated with active *B. abortus* infection.

The intensity of *B. abortus* infection was expected to increase during periods of food restriction, as body reserves were metabolized to meet competing nutritional demands. Culture results were reevaluated from actively infected bison (e.g., culture positive, *n *=* *94) to identify age-specific effects of nutrition and nutritional condition on the intensity of infection using logistic-binomial models. The response was the total number of culture positive plates observed for each individual bison. Infection intensity was defined as the probability of observing a culture positive result on a single plate, with the probability corresponding to covariates for bison age, nutrition, and nutritional condition using the inverse-logit transformation. Bison ≥7 years of age were grouped into a single old-adult age class. To maximize sample size, the effects of nutrition and nutritional condition were analyzed separately. A suite of 22 additive models was developed to assess the effects of nutritional condition (*n *=* *53 records) on infection intensity. The model suite included all combinations of age, the quadratic effect of age, live weight, back fat thickness, and body condition score. Because most Yellowstone bison become exposed to *B. abortus* as juveniles (Treanor et al. 2011), we predicted the intensity of infection would be greatest in younger ages (i.e., reproductively immature animals), with infection intensity negatively associated with age and covariates for nutritional condition.

We also developed a suite of 32 models to assess effects of nutrition (metabolites and hormones; *n *=* *78 records) on infection intensity. The model suite included all combinations of age, the quadratic effect of age, and concentrations of plasma indicators of nutrition (BUN, NEFA, IGF-1, and leptin). Again, we predicted the intensity of *B. abortus* infection to be greatest in young bison experiencing undernutrition. We expected infection intensity to be negatively associated with age, BUN, IGF-1 and leptin, but positively associated with NEFA. Maximum likelihood estimation was used to determine model parameter magnitudes using the package bbmle in program R (R Development Core Team 2010). Models were ranked using Akaike's information criterion corrected for small sample size (AIC_c_); normalized AIC_c_ weights (*w*_*i*_) were used to compare candidate models. Models with AIC_c_ differences (Δ_*i*_) ≤ 2 from the top supported model were considered to have substantial empirical support, while models with 4 ≤ Δ_*i*_ ≤ 7 have considerably less support, and models having values Δ_*i*_ ≥ 10 have essentially no support (Burnham and Anderson 2002). We examined 95% confidence intervals of model parameters to determine the relative magnitude and direction of effects. Normalized AIC_c_ weights (*w*_*i*_) were used to compare the fit of candidate models to the data.

## Results

### Season and age effects

**Figure 3:**
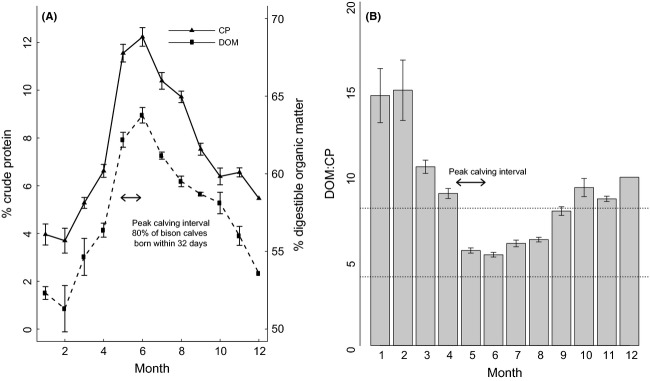
Monthly percentage (mean ± SE) of (A) fecal crude protein (CP) and digestible organic matter (DOM) and (B) DOM:CP ratio for bison in Yellowstone National Park. The calving interval for Yellowstone bison was identified in Jones et al. (2010). Bison calving is synchronized with the emergence of forage high in protein and digestible energy during spring. Dietary DOM:CP ratios between 4 and 8 indicate a favorable protein-energy balance to meet reproductive demands.

The analysis of bison fecal samples demonstrated a seasonal pattern in diet quality, with peak forage quality occurring immediately following spring vegetation green-up and extending into early summer. The period from May to June, which corresponds with the timing of bison calving and high lactation demands, had the highest percentage of crude protein and digestible organic matter (Fig.3A). The ratio of digestible organic matter to crude protein (DOM:CP) is a useful metric of rumen efficiency, with a range between 4 and 8 being acceptable and values closer to 4 being optimal (Odadi et al. 2011). The DOM:CP ratio was within the range expected for weight gain during late spring and early summer (May to July: 5.3–5.9), while the ratio during winter and early spring reflected low-quality forage (Jan to April: 8.8–14.5; Fig.3B).

**Figure 4:**
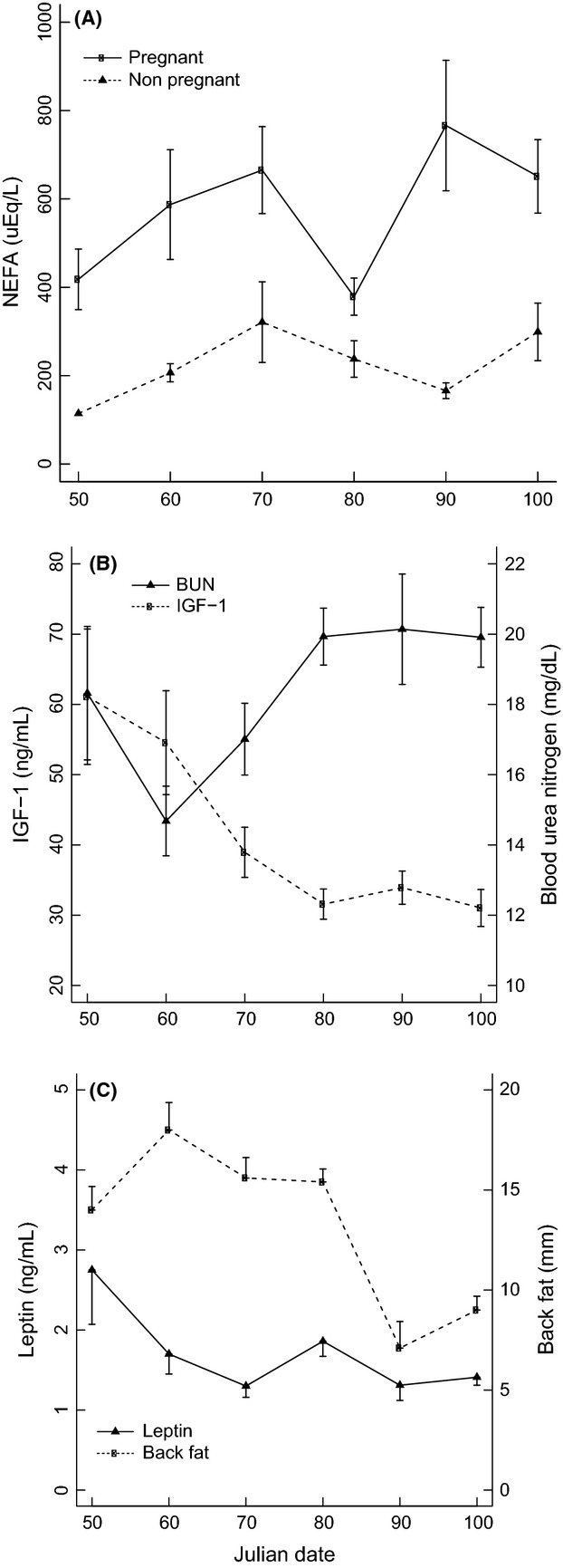
Differences in blood metabolites, hormones and body condition in female Yellowstone bison sampled during late gestation. Julian date refers to the numeric date starting on Jan 1, 2008 (e.g., 50 = Feb 9 and 100 = April 9). Values are presented as mean ± SE for concentrations of (A) nonesterified fatty acid (NEFA), (B) insulin-like growth factor-1 (IGF-1) and blood urea nitrogen (BUN), and (C) leptin and back fat measurements.

**Figure 5:**
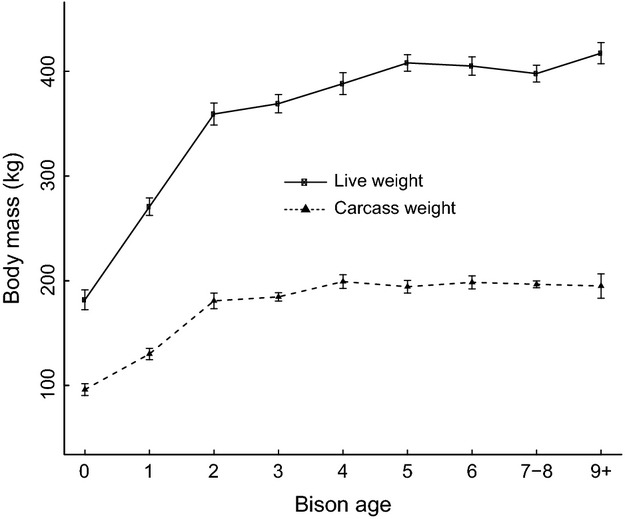
Differences in body mass (mean ± SE) with increasing age in female Yellowstone bison. Live weight represents measurements on intact animals, including pregnant and nonpregnant adults. Carcass weight represents measurements on processed animals without internal organs and reproductive tissues.

The body condition and metabolic and hormonal responses of pregnant bison sampled during late gestation (February 9 to April 9) implied a prolonged state of undernutrition (Fig.4). Mean concentrations of NEFA in pregnant bison increased during the sampling period and were greater than concentrations in reproductively mature nonpregnant bison (Fig.4A), indicating a shortage of dietary energy. The rise in BUN and corresponding decline in IGF-1 (Fig.4B) reflect a shortage in dietary protein that coincided with a decline in dietary energy. The mean concentration of plasma leptin and back fat thickness declined during late gestation (Fig.4C), further reflecting an increase in fat metabolism and a state of negative energy balance. Annual changes in bison live weight and carcass weight were significantly greater (*P *<* *0.001) in young bison below the age of three than in older individuals (Fig.5), which shows the investment in growth by juvenile bison.

**Figure 6:**
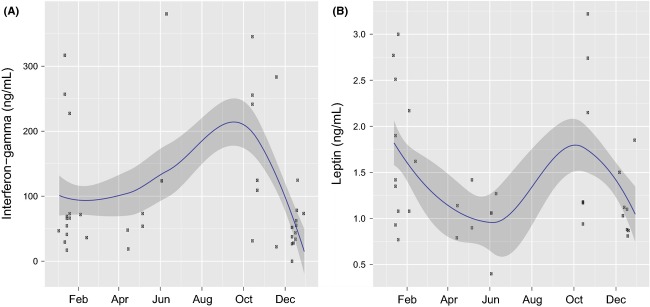
Scatterplot of (A) interferon-*γ* and (B) leptin in female Yellowstone bison sampled from 2007 to 2009. The interferon-*γ* levels were measured by ELISA from the culture supernatants of peripheral blood mononuclear cells stimulated by the mitogen Concanavalin A. The solid line is the LOESS smoother and the gray area represents the 95% confidence band.

The secretion of IFN-*γ* by stimulated cells (ConA) was greatest in bison sampled during autumn and reduced in animals sampled during winter or spring (Fig.6A). Plasma leptin concentrations were lowest in spring (Fig.6B), indicating a seasonal decline in energy reserves. Mean IFN-*γ* responses to unstimulated cells (media only) were near zero for all seasons measured (autumn = 0.08; winter = 0.09; and spring = 0.06 ng/mL IFN-*γ*), which demonstrates that stimulation of cells is necessary for measuring indicators of T-cell-mediated immunity (e.g., IFN-*γ*) in bison blood.

### Analysis of infection

**Figure 7:**
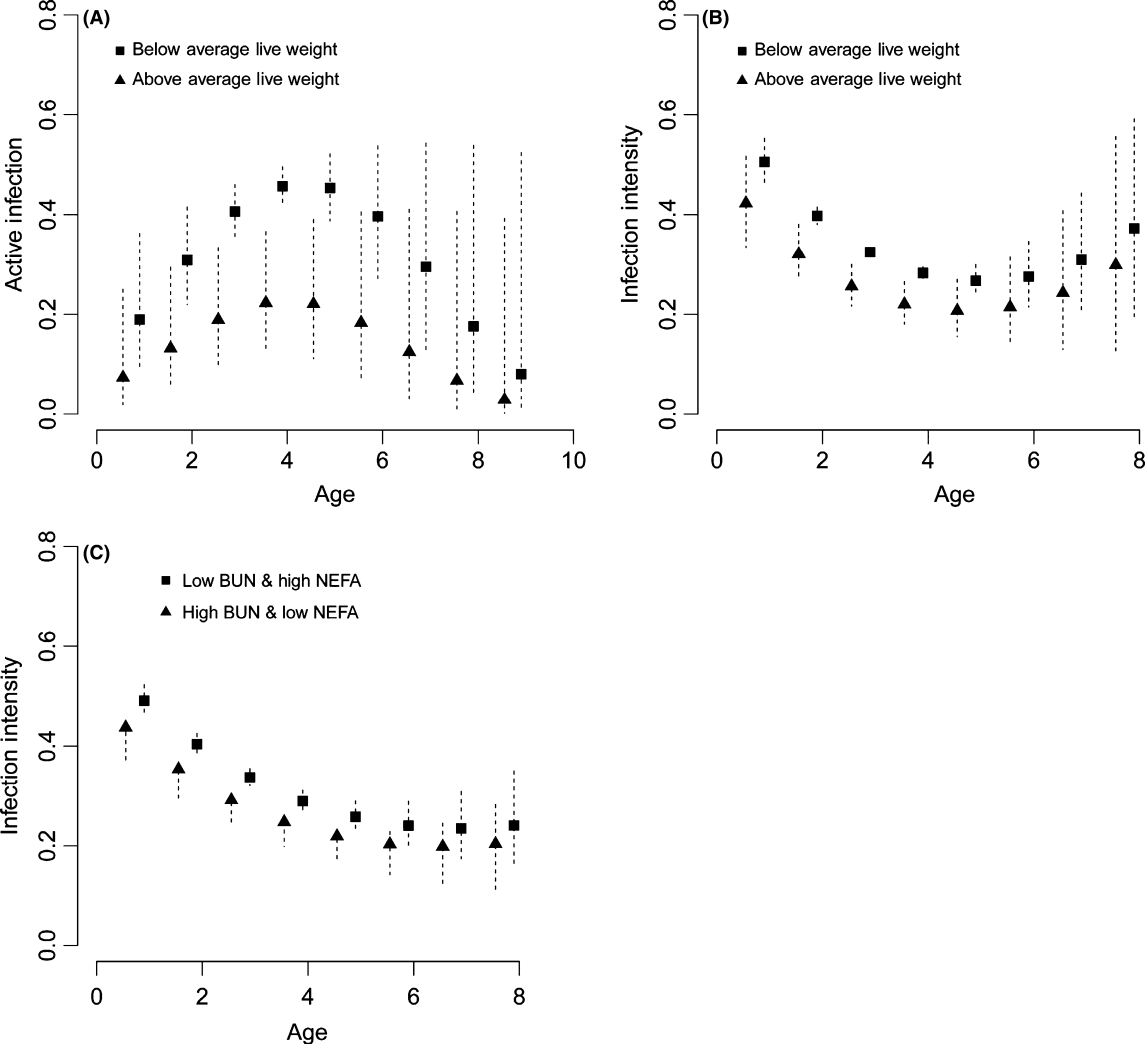
Predicted probabilities of active *Brucella abortus* infection and intensity of infection across bison age for the dependent variable (A & B) bison live weight and (C) concentrations of blood urea nitrogen (BUN) and nonesterified fatty acid (NEFA). Below- and above-average live weight and low and high BUN and NEFA represent data from the 20th and 80th percentile, respectively. The dashed lines indicate the 95% confidence limits for the predictions.

The probability of active brucellosis infection was influenced by age and age-specific live weight, with the highest probability observed in early reproducing bison (ages 3–5; Fig.7). The two top supported models (83% of *w*_*i*_; Table1a) included covariates for bison age, live weight, and back fat. However, the 95% confidence interval for the back fat coefficient included zero (Table2), which suggests that it did not improve model performance. Confidence limits of the coefficient estimates for bison live weight and age^2^ did not overlap zero and indicated these covariates were negatively associated with the probability of active infection. Fitting bison age as a quadratic covariate improved model fit by allowing for a curvilinear response, such that the probability of active infection could increase early in life, peak during early reproductive years, and decline with increasing age. Live weight had less support in models without age^2^ (ΔAIC_c_ = 4.43–4.97), while models without live weight had essentially no support (ΔAIC_c_ > 10; ∑*w*_*i*_ = 0). These findings suggest that bison in below-average body condition in this early reproducing age group had the highest probability of active brucellosis infection (Fig.7A).

**Table 1:** Ranking of regression models evaluating (a) active *Brucella abortus* infection (logistic) and the (b,c) intensity of infection (binomial-logistic) in Yellowstone bison. Models are ranked by ΔAIC_c_ and Akaike weights (*w*_*i*_)

Model	*K*	AIC_c_	ΔAIC	*w*_*i*_
(a) Active Infection				
Age + Age^2^ + LW + BF	5	155.45	0.00	0.43
Age + Age^2^ + LW	4	155.61	0.16	0.40
LW	2	159.88	4.43	0.05
LW + BF	3	160.17	4.72	0.04
Age + LW	3	160.38	4.93	0.04
Age + LW + BF	4	160.42	4.97	0.04
(b) Infection Intensity – Condition				
Age + Age^2^ + LW	4	561.74	0.00	0.32
Age + Age^2^ + LW + BCS	5	561.76	0.02	0.31
Age + Age^2^ + LW + BCS + BF	6	562.39	0.65	0.23
Age + Age^2^ + LW+ BF	5	564.18	2.44	0.09
(c) Infection Intensity – Nutrition				
Age + Age^2^ + BUN + NEFA + IGF-1 + LEP	7	754.23	0.00	0.34
Age + Age^2^ + BUN + NEFA + LEP	6	754.41	0.18	0.31
Age + Age^2^ + BUN + NEFA	5	756.16	1.93	0.13
Age + Age^2^ + BUN + NEFA + IGF-1	6	757.05	2.82	0.08
Age + Age^2^ + BUN + LEP	5	758.02	3.79	0.05
Age + Age^2^ + BUN + IGF-1 + LEP	6	758.34	4.11	0.04
Age + Age^2^ + BUN	4	759.75	5.52	0.02
Age + Age^2^ + BUN + IGF-1	5	760.97	6.74	0.01

Model notation: LW, bison live body weight; BF, back fat thickness; BCS, body condition score; BUN, blood urea nitrogen; NEFA, nonesterified fatty acid; IGF-1, insulin-like growth factor-1; and LEP, leptin.

**Table 2:** Parameter estimates of top supported models (logistic) evaluating active *Brucella abortus* infection in female bison in Yellowstone National Park (*n *=* *152). For estimates not in bold, the 95% confidence interval includes zero

Active infection	Intercept	Age	Age^2^	Live WT	Back fat
Age + Age^2^ + LW + BF	−**0.68 (**−**1.22,** −**0.15)**	−0.56 (−1.41, 0.28)	−**1.82 (**−**3.29,** −**0.35)**	−**6.84 (**−**10.85,** −**2.84)**	−0.78 (−1.81, 0.24)
Age + Age^2^ + LW	−**0.69 (**−**1.22,** −**0.16)**	−0.50 (−1.34, 0.33)	−**1.77 (**−**3.21,** −**0.32)**	−**7.65 (**−**11.52,** −**3.78)**	
LW	−**1.19 (**−**1.59,** −**0.78)**			−**7.50 (**−**11.28,** −**3.71)**	
LW + BF	−**1.19 (**−**1.60,** −**0.78)**			−**6.74 (**−**10.67,** −**2.81)**	−0.67 (−1.67, 0.32)
Age + LW	−**1.17 (**−**1.57,** −**0.76)**	−0.44 (−1.14, 0.25)		−**7.16 (**−**10.93,** −**3.39)**	
Age + LW + BF	−**1.17 (**−**1.58,** −**0.76)**	−0.49 (−1.19, 0.22)		−**6.31 (**−**10.24,** −**2.39)**	−0.73 (−1.74, 0.28)

Model notation: LW, bison live body weight and BF, back fat thickness.

In bison actively infected with *B. abortus*, the intensity of infection was influenced by age and live weight (Fig.7B). There was strong support for models that included live weight, age, and age^2^ (∑*w*_*i*_ = 0.95; Table1b). The coefficient estimates for back fat thickness and body condition score were unstable. For these covariates, the direction of the effect varied between models and the 95% confidence interval for the estimated coefficients overlapped zero (Table3). Thus, back fat thickness and body condition scores were not found to be important predictors of infection intensity.

**Table 3:** Parameter estimates of top supported models (binomial-logistic) evaluating the influence of age and nutritional condition on *Brucella abortus* infection intensity in Yellowstone bison (*n *=* *53). For estimates not in bold, the 95% confidence interval includes zero

Infection intensity	Intercept	Age	Age^2^	LW	BF	BCS
Age + Age^2^ + LW	−**1.13 (**−**1.3,** −**0.96)**	−**0.38 (**−**0.61,** −**0.14)**	**0.60 (0.24, 0.95)**	−**1.82 (**−**2.85,** −**0.79)**		
Age + Age^2^ + LW + BCS	−**1.13 (**−**1.30,** −**0.96)**	−**0.37 (**−**0.6,** −**0.13)**	**0.58 (0.22, 0.93)**	−**1.65 (**−**2.70,** −**0.60)**		−0.04 (−0.90, 0.10)
Age + Age^2^ + LW + BCS + BF	−**1.13 (**−**1.30,** −**0.96)**	−**0.38 (**−**0.61,** −**0.14)**	**0.57 (0.21, 0.93)**	−**1.91 (**−**3.03,** −**0.79)**	−**0.69 (**−**1.33,** −**0.04)**	0.31 (−0.13, 0.76)
Age + Age^2^ + LW + BF	−**1.13 (**−**1.30,** −**0.96)**	−**0.38 (**−**0.62,** −**0.14)**	**0.6 (0.24, 0.95)**	−**1.84 (**−**2.96,** −**0.72)**	0.02 (−0.33, 0.37)	

Model notation: LW, bison live body weight; BF, back fat thickness; and BCS, body condition score.

For models assessing the influence of bison nutrition on the intensity of infection, the top supported models (ΔAIC_c_ < 3) included covariates for age, age^2^, BUN, and NEFA (∑*w*_*i*_ = 0.86; Table1c). The ranking of these models suggests that the intensity of *B. abortus* infection is greatest in young bison experiencing undernutrition, as indicated by lower levels of BUN and higher levels of NEFA (Table4). Leptin was also found to be an important predictor of infection intensity, but the positive direction of the effect was unexpected (Table4). Coefficient estimates for IGF-1 suggest that the plasma concentration of this hormone is not an important predictor of infection intensity, as the estimated confidence interval included zero (Table4). These findings suggest that the intensity of *B. abortus* infection in actively infected bison corresponds with a reduction in dietary protein and an increase in fat metabolism that is most pronounced in young animals (Fig7C).

**Table 4:** Parameter estimates of top supported models (binomial-logistic) evaluating the influence of age and nutrition on *Brucella abortus* infection intensity in Yellowstone bison (*n *=* *78). For estimates not in bold, the 95% confidence interval includes zero

Infection intensity	Intercept	Age	Age^2^	BUN	NEFA	IGF-1	LEP
Age + Age^2^ + BUN + NEFA + IGF-1 + LEP	−**1.09 (**−**1.24,** −**0.94)**	−**0.55 (**−**0.70,** −**0.40)**	**0.32 (0.15, 0.48)**	−**0.88 (**−**1.30,** −**0.45)**	**0.17 (0.04, 0.30)**	−0.21 (−0.47, 0.05)	**0.26 (0.04, 0.48)**
Age + Age^2^ + BUN + NEFA + LEP	−**1.08 (**−**1.23,** −**0.93)**	−**0.56 (**−**0.70,** −**0.41)**	**0.31 (0.15, 0.47)**	−**0.71 (**−**1.08,** −**0.34)**	**0.16 (0.03, 0.29)**		**0.23 (0.01, 0.44)**
Age + Age^2^ + BUN + NEFA	−**1.08 (**−**1.23,** −**0.93)**	−**0.57 (**−**0.72,** −**0.43)**	**0.32 (0.15, 0.48)**	−**0.71 (**−**1.08,** −**0.34)**	**0.16 (0.03, 0.29)**		
Age + Age^2^ + BUN + NEFA + IGF-1	−**1.09 (**−**1.24,** −**0.94)**	−**0.57 (**−**0.71,** −**0.42)**	**0.32 (0.16, 0.49)**	−**0.83 (**−**1.25,** −**0.41)**	**0.17 (0.04, 0.29)**	−0.16 (−0.41, 0.10)	
Age + Age^2^ + BUN + LEP	−**1.07 (**−**1.22,** −**0.92)**	−**0.55 (**−**0.70,** −**0.41)**	**0.30 (0.14, 0.47)**	−**0.69 (**−**1.06,** −**0.32)**			**0.22 (0.01, 0.44)**
Age + Age^2^ + BUN + IGF-1 + LEP	−**1.08 (**−**1.23,** −**0.93)**	−**0.55 (**−**0.70,** −**0.40)**	**0.31 (0.15, 0.47)**	−**0.83 (**−**1.26,** −**0.41)**		−0.19 (−0.44, 0.07)	**0.25 (0.03, 0.47)**
Age + Age^2^ + BUN	−**1.07 (**−**1.21,** −**0.92)**	−**0.57 (**−**0.71,** −**0.42)**	**0.31 (0.15, 0.47)**	−**0.69 (**−**1.06,** −**0.32)**			
Age + Age^2^ + BUN + IGF-1	−**1.07 (**−**1.22,** −**0.92)**	−**0.57 (**−**0.71,** −**0.42)**	**0.32 (0.15, 0.48)**	−**0.79 (**−**1.21,** −**0.37)**		−0.13 (−0.38, 0.12)	

Model notation: LW, bison live body weight; BF, back fat thickness; BUN, blood urea nitrogen; NEFA, nonesterified fatty acid; IGF-1, insulin-like growth factor-1; and LEP, leptin.

## Discussion

The objective of this study was to evaluate a conceptual framework that links seasonal food insufficiency with *B. abortus* infection based on how nutritional resources are allocated across bison life stages. Female bison showed signs of undernutrition during late gestation when diet quality was low, while bison calving coincided with the emergence of highly nutritious spring forage needed to meet increasing reproductive demands. Active infection was negatively associated with age and nutritional condition in female bison. For infected animals, the intensity of *B. abortus* infection was related to indicators of dietary protein and energy restriction and was most pronounced in juvenile bison. A successful strategy for *B. abortus* is to maintain a large reservoir of infected juveniles that will develop into the major transmission source in their early reproductive years. Additionally, the immune response (IFN-*γ*) in bison was highest during autumn, when animals are in good nutritional condition, and reduced in winter and spring. Our results suggest that the infectivity of *B. abortus* is associated with the nutritional state of Yellowstone bison in a dynamic environment, and the investment of limited resources toward reproduction over immune defense may be an important trade-off influencing infection probabilities in young bison.

### Seasonal nutrition and nutritional condition

Our results support the hypothesis that seasonal changes in forage quality influence the reproductive cycle in Yellowstone bison by influencing the timing of parturition. There is a strong relationship between plant phenological stages and the nutritional quality of herbivore diets, with energy and protein content generally being highest in early plant growth stages (Van Soest 1994; Mysterud et al. 2001). As expected, fecal crude protein and digestibility were highest in late spring through early summer and declined to the lowest levels during winter and early spring (Fig.3A). The emergence of high-quality spring forage may explain the synchrony of calving observed in Yellowstone bison (Rutberg 1984). High-quality forage at the onset of the calving period through the breeding season has fitness benefits for bison and other ungulates by increasing calf survival and allowing postpartum females to replenish body reserves sufficiently to initiate their next reproductive cycle (Cook et al. 2013).

Yellowstone bison are also challenged to meet their maintenance and energy needs during winter as dietary protein and energy decline below maintenance levels (DelGiudice et al. 2001). In wild adult ruminants, the level of crude protein necessary to maintain minimum protein balance ranges from 5 to 9% (Parker et al. 2005). In our study, bison fecal analysis suggested that forage crude protein was below maintenance levels during the winter (Fig.3A). The percentage of digestible organic matter, an indicator of available energy, also declined over the growing season as the amount of indigestible structural tissues increased in growing plants. Ruminal microbes need a balanced supply of dietary energy and protein, with a ratio of 4:1 being ideal (McCollum 1997). The ratio of digestible organic matter to crude protein (DOM:CP) indicated that bison had access to high-quality forage from May to August (Fig.3B), which coincides with lactation demands and replenishment of body reserves needed for ovulation in late summer. However, forage quality was well outside this range during the winter months, the interval of increasing fetal demands in late gestation. The reproductive strategy in Yellowstone bison links calving with the availability of high-quality forage, but also ensures that pregnant females are in poor condition when forage quality is low in winter and early spring.

In temperate and northern ecosystems, the effect of seasonal food restriction on wild animals can be better understood by indicators (e.g., metabolites and hormones) that have specific links to nutritional status (Karasov and Martínez del Rio 2007). For ruminants, peak energy requirements coincide with late gestation and early lactation (Van Soest 1994), which are met by the mobilization of fat reserves. As food restriction and pregnancy advance, nonesterified fatty acid (NEFA) levels rise, indicating a shortage in dietary energy relative to fetal energy demands (Yambayamba et al. 1996). Pregnant bison had elevated plasma NEFA concentrations 2–3 times that of mature, barren females (Fig.4A), indicating a state of negative energy balance during late gestation. Observed increases in blood urea nitrogen (BUN) and declines in insulin-like growth factor-1 (IGF-1; Fig.4B) indicate that pregnant bison were also experiencing a reduction in protein nutrition during late gestation. Protein restriction decreases BUN; however, during a long period of negative energy balance, muscle tissue is catabolized as a gluconeogenic substrate and BUN increases (Harder and Kirkpatrick 1994; Karasov and Martínez del Rio 2007). Additionally, the endocrine hormone IGF-1 is sensitive to dietary protein with reduced levels signifying periods of deficient protein and calorie ingestion (Gomes et al. 2003). The decline in plasma leptin and back fat thickness during late gestation (Fig.4C) reflect an increase in fat metabolism and an advancing state of negative energy balance. Leptin, a peptide hormone secreted primarily by adipose tissue, tracks available energy stores (fat) and has been linked to fat metabolism and energy balance in mammals (Chilliard et al. 2005; Spady et al. 2009). Further, the ability to communicate the status of fat stores makes leptin an attractive candidate for modulating immune responses in accordance with energetic reserves (Adelman and Martin 2009; Borghetti et al. 2009; French et al. 2009). Cumulatively, these findings support the hypothesis that food restriction during winter reduces fat and protein stores in Yellowstone bison, which contribute to a decline in nutritional condition.

### Active infection and intensity of infection

Active brucellosis infection in female bison was negatively associated with bison age and nutritional condition. Infection probabilities increased during prereproductive years, peaked in early reproductive ages (2–4 years old), and declined with increasing age. For each age class, the probability of isolating *B. abortus* was greatest for animals in below-average condition (Fig.7A). While the significant effect of reduced body weight suggests nutritional stress increases susceptibility to infection, the data were not obtained through experimental manipulation. Therefore, we cannot rule out that active *B. abortus* infection may have caused the observed reduction in body condition, especially if young bison are mounting costly immune responses against infection. In many cases, nutritional condition can decline within a host as infection progresses and the cost of parasitism increases. However, this is likely not the case for bovine brucellosis, as most bovids do not suffer marked fever, anorexia, or other signs of disease when infected (Cheville et al. 1998). We found that across age groups the majority of bison in below-average nutritional condition were not actively infected with *B. abortus* because declines in nutritional condition over winter are independent of infection. There was no significant difference (*P* < 0.05) in the most important indicators of condition (Live Weight) and nutrition (NEFA and BUN) between infected (culture positive) and noninfected (culture negative) bison for the main age groups (0–2 years old, 3–6 years olds, & 7+ years olds). However, age and host body mass were important factors that were negatively associated with the probability of active infection. Additionally, susceptibility to infection is known to be associated with sexual maturity (Thorne 2001), which is consistent with the probability of active infection being greatest in female bison in early reproductive ages.

*Brucella abortus* is able to establish persistent infections because it has evolved mechanisms to suppress innate immune responses in the host during early infection (Spera et al. 2006; Carvalho Neta et al. 2008). The strategy of *B. abortus* is to remain undetected and replicate to large numbers in placental cells during late gestation when the immune system of the pregnant host is naturally suppressed. Therefore, juvenile bison are unlikely to experience costly (proinflammatory) immune responses to infection because they are not yet sexually mature. Based on the pathogenesis of *B. abortus* in ruminants, the association between nutritional condition and active infection is more consistent with increased vulnerability to infection in young bison rather than the result of infection. Nevertheless, an experimental study could be conducted to conclusively determine the extent to which nutritional condition increases susceptibility to *B. abortus* infection in bison.

Loss of body mass may be the critical factor that mirrors compromised immune function (Nelson et al. 2002). For immature bison, prioritizing body reserves for maintenance and growth over immune defense could be the best strategy for survival and reproduction. Survivorship increases with body size in juvenile ungulates (Parker et al. 2009), while nutritional restriction in juveniles can increase the age at first reproduction and reduce lifetime reproductive success (Gaillard et al. 2000). Over-investment in immune defense may result in fitness costs, such as age and size at maturity (Sorci et al. 2009). Despite the ability to avoid host immune responses, approximately 90% of *Brucella* bacteria are killed within the first few hours after entering their host (Skendros and Boura 2013; Olsen and Palmer 2014). However, *B. abortus* may pose little threat to the immune defenses of immature hosts, obviating costly immune responses. The prevalence of active *B. abortus* infection in undernourished juvenile bison may reflect a trade-off in immune defense for growth and maintenance, which could increase survival and future reproductive success.

Individual bison life stages can represent different habitats for *B. abortus*. In our study, the intensity of infection was negatively associated with nutrition and body condition in juveniles and bison at early reproductive ages, which implies that young bison in below-average condition harbor the highest densities of *Brucella* bacteria. Malnourished juveniles may be vulnerable to infection but are a dead-end host for *B. abortus* transmission until females reach sexual maturity and become pregnant. In long-lived ungulates, such as bison, young females reproduce before reaching their adult weight and often bear the energetic costs of growth and lactation simultaneously (Hamel et al. 2010). Primiparous bison may have compromised immune function during the *B. abortus* transmission period because they are still growing, and body reserves are largely depleted during late gestation. In older bison, the decline in active infection may indicate some level of acquired immunity after experiencing acute infection earlier in life (Treanor et al. 2011). Our findings suggest that active *B. abortus* infection is influenced by nutritional condition, with seasonal food restriction assisting infection of immature bison, which become the primary transmission source during early reproductive ages.

When food is limited, mounting an effective immune response to contain and overcome parasitic infections can have considerable nutritional costs (Valderrábano et al. 2006; Martin et al. 2008). Consequently, hosts in poor condition might have higher infection intensities because parasites would encounter less opposition to their survival and proliferation (Beldomenico and Begon 2010). Our results suggest that infection intensities are exacerbated by seasonal reductions in dietary protein and energy. The association of BUN and NEFA levels with infection intensities indicates that reduced protein intake and elevated fat metabolism (i.e., energy needs) may increase susceptibility to *B. abortus* infection. Deficiencies in dietary protein increased susceptibility to gastrointestinal parasites in wild bovids (Ezenwa 2004), while reductions in total body fat reduced humeral immunity in rodents (Demas et al. 2003). Because *B. abortus* is an intracellular parasite, immune defense requires effective T-cell-mediated responses which have high costs in terms of protein and energy (Calder and Jackson 2000; Buehler et al. 2010). Further, the activation of peripheral blood mononuclear cells (PBMC) is needed to defend against *B. abortus*; however, PBMC activation is strongly dependent on energy availability and, thus, might be compromised in situations with competing energy requirements, such as pregnancy and lactation (Wyckoff 2002, Schwarm et al. 2013).

The positive association between leptin and the intensity of *B. abortus* infection in bison was unexpected. Leptin functions as a signal in a feedback loop regulating food intake and body weight and has been found to be correlated with the amount of fat tissue. Leptin levels and back fat thickness indicated bison were in a state of negative energy balance; we therefore expected higher infection intensities in animals with lower plasma leptin. However, leptin levels in bison were comparable to those in food-restricted animals (Delavaud et al. 2000; Soppela et al. 2008) and may have signaled that energy reserves were depleted. The positive correlation between leptin and the amount of subcutaneous fat (back fat) was fairly low (*r* = 0.22), which suggests that leptin was not a strong predictor of back fat thickness in the sampled bison. We found that NEFA (indicator fat metabolism) and BUN (indicator of protein intake) were the most important indicators of nutrition in relation to infection intensity (Table1c).

Leptin plays an important role in the generation and maintenance of T-cell responses (e.g., T_helper_ 1), which are reduced at low leptin levels (Lord 2002; Bernotiene et al. 2006). As a result, T-cell responses in bison may have been down-regulated as leptin levels communicate the amount of energy available for the immune system (French et al. 2011). As a mediator of the inflammatory immune response, leptin increases the production of IFN-*γ* (Fernández-Riejos et al. 2010), which plays a major role in protection against *B. abortus* (Clapp et al. 2011). The reduction of IFN-*γ* and leptin during spring (Fig.6A and B) suggests that immune defenses against *B. abortus* may be suppressed in free-ranging Yellowstone bison during the primary transmission period.

While it is well-established that *B. abortus* can be transmitted within wild ruminant populations or domestic livestock in good nutritional condition, susceptibility to infection at the individual level is known to be based on age, stress factors, and probably some level of genetic resistance (Cheville et al. 1998; Thorne 2001). The reduction in nutritional condition caused by seasonal food restriction may also influence *B. abortus* infection, especially in young animals. In Yellowstone bison, the prevalence of *B. abortus* infection fluctuates across years. Some of this annual variation may be attributed to the severity of winter conditions that restrict food availability and reduce the nutritional condition of wild bison. Therefore, our findings are relevant to brucellosis dynamics in wildlife populations in the Greater Yellowstone Area and other temperate systems with *B. abortus* infected wildlife.

Some caution needs be applied to the interpretation of our analyses because of the cross-sectional, correlative nature of data collected from slaughtered animals. Typically, longitudinal data would be needed to understand the association between nutrition, body condition and active *B. abortus* infection in free-ranging bison. Yet, longitudinal data cannot be used to estimate active *B. abortus* infection and the intensity of infection because that information can only be obtained from dead animals. Currently, a longitudinal study involving repeated measurements on live animals would be restricted to serologic tests (blood tests), which cannot determine true infection from exposure (antibody presence) and are incapable of quantifying infection intensities. Also, important indicators of nutritional condition, such as live weight, require capture facilities with scales to weigh large mammals. Therefore, wild bison would need to be repeatedly corralled into facilities, which may not be realistic, especially for wildlife preserves. Nevertheless, our results support the hypothesis that active *B. abortus* infection and the intensity of infection is influenced by the age and nutritional condition of the bison host.

## Conclusions

Our findings have application to management efforts attempting to reduce the level of *B. abortus* in bison and elk in the greater Yellowstone ecosystem, as well as bison in the Wood Buffalo area of Canada (Nishi 2010). During spring, Yellowstone bison seek emerging forage on low elevation ranges outside the park but are frequently pushed back onto high elevation ranges within the park where snow cover delays the growth of new vegetation. This management approach maintains separation between Yellowstone bison and cattle outside the park and has been successful at preventing *B. abortus* transmission from bison to cattle. However, management practices that prevent bison from accessing nutritious food during the *B. abortus* transmission period may contribute to the maintenance of brucellosis in Yellowstone bison. Extending the period of food restriction when reproductive demands are greatest and body reserves are depleted is expected to further suppress immune function. Management practices that concentrate bison in the park during the *B. abortus* transmission window increase transmission potential as bison densities increase near park boundaries. Conversely, management practices that improve the nutritional condition of Yellowstone bison may improve the ability of bison to respond to *B. abortus* infection and also increase the effectiveness of vaccines aimed at reducing transmission.

The overlap of seasonal food restriction with the high nutritional demands of reproduction in young bison may be an important occurrence sustaining the prevalence of *B. abortus* infection. Such recurrent environmental conditions favor *B. abortus*, which can establish persistent infections in juveniles. In adults, synchronizing calving with the emergence of spring forage is an effective strategy for meeting nutritional costs of lactation and increasing calf survival (Rutberg 1987). However, this strategy requires pregnant bison to be in a state of reduced nutritional condition at a time when food is limited and reproductive demands of late gestation are elevated. The high prevalence of active infection in juvenile bison may reflect greater exposure risk that results from social interactions with infectious pregnant females during the calving season (Treanor et al. 2011). These conditions may facilitate *B. abortus* transmission when infected juveniles reach sexual maturity. The endemicity of brucellosis in Yellowstone bison might be a consequence of the vulnerability of young animals to infection and the concurrence of seasonal food limitation with the reproductive needs of pregnant females. Our findings contribute to the understanding of how physiological trade-offs might drive seasonal immune suppression, which influences infection heterogeneities in wildlife populations.
